# The Golden Card of Interleukin-1 Blockers in Systemic Inflammasomopathies of Childhood

**DOI:** 10.3390/ijms26051872

**Published:** 2025-02-21

**Authors:** Donato Rigante

**Affiliations:** 1 Department of Life Sciences and Public Health, Fondazione Policlinico Universitario A. Gemelli IRCCS, 00168 Rome, Italy; donato.rigante@unicatt.it; Tel.: +39-06-30155210; 2 Periodic Fever and Rare Diseases Research Centre, Università Cattolica Sacro Cuore, 00168 Rome, Italy

**Keywords:** autoinflammatory disorder, autoinflammation, periodic fever, personalized medicine, interleukin-1, anakinra, rilonacept, canakinumab, innovative biotechnologies, pediatrics, child

## Abstract

A growing number of systemic hereditary inflammatory diseases characterized by periodic fevers and elevated acute-phase proteins during flares has been linked to deregulated inflammasome function and excessive bioactivity of interleukin (IL)-1. All these conditions respond, at varying degrees, to the specific blockade of IL-1. The remarkable progress with IL-1 antagonists in treating hereditary inflammasome-based disorders has offered new hope for several patients with further non-hereditary autoinflammatory conditions from multifactorial backgrounds. The effectiveness of the IL-1 blockade has transformed our understanding and management of many complex diseases and highlighted the role of aberrant IL-1 signaling in enigmatic conditions, characterized by recurrent or continuous inflammation and a lack of a role for autoreactive T-cells or autoantibody production. To date, the long-term blockade of IL-1 has been found to restore the clinical equilibrium in systemic inflammasomopathies of childhood, and IL-1 inhibitors have become cardinal weapons in managing both monogenic innate immunity defects and a plethora of polygenic diseases occurring in children, including Still’s disease, Kawasaki disease, recurrent pericarditis, chronic non-bacterial osteomyelitis, and Behçet’s disease. Very few side effects have been reported with the long-term use of anakinra, rilonacept, or canakinumab, and their safety profile has been largely documented even in childhood. Further investigations into the role of inflammasomes in the pathogenesis of autoimmune conditions as well as brain degenerative or cardiovascular disorders can be expected, paving the way for precision medicine with benefits beyond inhibiting signaling by individual IL-1-family cytokines.

The human immune system is equipped with a multi-layered *gendarmerie* to sense and counteract infectious agents, endogenous metabolites, or non-self and hidden dangers with the aim of ensuring the body’s safety and its integrity [1]. Immunity cells impact the response gating through pattern recognition receptors which coordinate cell migration, phagocytosis, cytolytic activity, and the release of cytokines to patrol and manage cell turnover and death in jeopardized tissues [2]. Interleukin (IL)-1 is an ancestral pyrogen with varying functions through a squad of adjutants, governing an intertwined network of molecules which *incipiunt* many innate immunity responses after a stressful event [3]. The components of the ‘enlarged’ IL-1 family include ligands endowed with agonist, antagonist, pro-inflammatory, or anti-inflammatory activities, and receptor chains that contribute to the shaping and steering of inflammation, as well as polarizing myeloid cells towards either innate or adaptive pathways: IL-1α,/1β, IL-18, IL-33, IL-36α/β/γ act as extracellular soluble factors, while IL-1/IL-36 receptor antagonists, IL-37, IL-38, with uneven powers and localization, have counterbalancing effects [4]. IL-1α and IL-1β share the same receptor complex, and both are synthesized as outrider proteins: the first by mesenchymal cells without the need for enzymatic cleavage, and the second by different mononuclear cells after caspase-related cleavage [5]. IL-18 is another IL-1 family-striker which induces interferon release and promotes cytotoxic and natural killer cells, magnifying the loop of IL-1 activities, and finally enhancing antimicrobial activity and long-term immunologic memory [6]. Both IL-1β and IL-18 are generated by the ‘inflammasome’, an intracellular multi-protein complex that transmutes the sensing of danger signals to caspase-1 cleaving activity upon pro-IL-1β and pro-IL-18 [7]. According to the dominant driver of inflammation—specifically IL-1, nuclear factor-κB [combined with tumor necrosis factor (TNF)], or interferon—the related “autoinflammatory diseases” can be outlined into inflammasomopathies, relopathies, or interferonopathies, respectively, justifying coherently targeted therapies (Table 1).

Inflammasome activation triggers two major responses: the induction of gasdermin D pore-mediated inflammatory cell death, known as *pyroptosis*, and the caspase-1-promoted secretion of pro-inflammatory cytokines. The subverted production of IL-1 bonds many conditions driven by disrupted inflammasome activity [8], either monogenic or multifactorial, that may involve countless tissues and organs, even the seemingly most inert ones like serosal membranes and skin [9,10]. Inflammasomes take their name from the sensor component; of these, the NLRP3 inflammasome is the most studied: the discovery that gain-of-function *NLRP3* mutations caused the cryopyrin-associated periodic syndrome (CAPS) was a breakthrough at the doorway of the post-genomic medicine era [11]. Non-anti-IL-1 agents, which were the first biologics introduced in pediatric rheumatology, are poorly effective in controlling the CAPS disease spectrum, varying from familial cold-induced urticaria to CINCA syndrome [12], while IL-1 inhibition was the only solution for the most severe CAPS symptoms: initially anakinra, the recombinant form of the natural IL-1 antagonist that inhibits both IL-1α and IL-1β, followed by rilonacept, a dimeric fusion protein consisting of the soluble receptor that traps both IL-1α and IL-1β, and canakinumab, the human-specific anti-IL-1β monoclonal antibody [13,14].

A peculiar pyrin inflammasome is otherwise involved in children with familial Mediterranean fever (FMF), the most common autoinflammatory disorder, which is caused by mutations in the *MEFV* gene. Mutated pyrins specifically have a decreased activation threshold, leading to pyrin hypophosphorylation and blooming release of IL-1β and IL-18 [15,16]. The link to IL-1 production may be less direct for other conditions, such as TNF receptor-associated periodic fever syndrome, caused by mutations in the *TNFRSF1A* gene encoding the 55-kD TNF receptor: neutrophils of these patients are less prone to apoptosis and oversecrete many transcription factors [17], but the intracellular accumulation of misfolded TNF receptors triggers inflammasome activation and enhances IL-1β release [18]. Lastly, the increased production of IL-1β due to reduced protein geranylgeranylation underlies the inflammatory flares in children with mevalonate kinase deficiency, a metabolic disease arising from variants within the *MVK* gene, which block isoprenoid biosynthesis and bring about a vast array of autoinflammatory manifestations [19].

For these reasons, the IL-1 pathway is the logical therapeutic target for patients affected by inflammasomopathies, as supported by different case series demonstrating the efficacy of IL-1 inhibitors, confirmed by improvements in inflammatory markers and improvements in diary scores, as well as physician and patient global pain assessment tools. Despite their motley clinical signs, heritability pattern, and penetrance, all inflammasomopathies have been shown to share a favorable response to the IL-1 blockade, which has become their seminal branding. A diagnosis of inflammasome-related diseases may even be corroborated by the results following the administration of IL-1 inhibitors [20]. The once-daily injection of anakinra, the once-weekly injection of rilonacept, and the once-monthly injection of canakinumab may result in a different compliance to therapies. However, there are very few randomized controlled studies assessing interventions for children with inflammasomopathies comparing active interventions (including IL-1 antagonists) with placebo or no treatment or comparing active drugs to each other. 

In addition to notable success in treating patients with such rare autoinflammatory diseases, targeting IL-1 activity has shown considerable promise in more common diseases considered to have inflammatory IL-1 activity as a key pathogenic mechanism. For instance, this strategy has been extended beyond pediatric patients to include brain degenerative or cardiovascular disorders and autoinflammatory-based pericardial diseases, while the significance of IL-1 in tumor development is currently under investigation [21]. Despite the clinical advantage of IL-1 blockers over other standards of care, they unfortunately remain largely out of reach for patients living in low- or middle-income countries, reflecting access to care as an arduous social issue that is prevalent worldwide [22]. In addition, even if IL-1 blockers have presented excellent long-term effectiveness in terms of drug retention rate, there are no strict rules to support the choice of a specific drug, and no recommendations are available for dose escalations.

The overexpression of inflammatory cytokines, such as IL-1, has led to the consideration of other complex polygenic conditions in childhood marked by an oversensitive innate immune system with loss of a physiological regulation. Indeed, recipients of the IL-1 blockade have expanded to include many other children affected by protean diseases in which monocytes, macrophages, and neutrophils are the main dysfunctional cells [23]. For instance, Still’s disease is an infrequent hyper-cytokinemic syndrome characterized by fever, skin rash, generalized lymphoid hyperplasia, thrombocytosis, and huge levels of ferritin; it also exhibits life-threatening complications such as macrophage activation syndrome and chronic lung disease, for which IL-1 inhibition has shown the highest therapeutic efficacy [24]. Despite the small number of randomized controlled trials with IL-1 inhibitors, they are strongly recommended in the treatment of Still’s disease due to the overwhelming body of evidence from real-world experience supporting their efficacy in controlling all aspects of the disease and to limit patients’ exposure to corticosteroids. Increasing evidence has also revealed the central role of IL-1 signaling in cases of coronary arteritis in children with Kawasaki disease, a *bizarre* disease occurring worldwide, but predominantly in East Asian children, for which IL-1 dysregulation parallels the severity of systemic inflammation and treatment responsiveness [25,26]. IL-1 antagonists have also been essential for the full management of a recently reported hyper-cytokinemic condition, i.e., coronavirus-related multisystem inflammatory syndrome [27]. Interestingly, the investigation into such phenotypes has further disclosed the coexistence of innate and adaptive immune abnormalities, blurring the thin line between autoinflammation and autoimmunity [28].

As the biological pipeline advances, the discovery of additional novel pathways that are probably involved in the pathogenesis of autoinflammatory disorders might provide more personalized and tailored solutions. For now, both observational studies and clinical trials have certified the amazing efficacy of the IL-1 blockade via anakinra, rilonacept, and canakinumab in cases of inflammasomopathy, also confirming their excellent safety profile in children [29]. A sustained abatement in the severity of inflammasomopathies might depend on the decision to start IL-1 inhibitors as soon as possible. Future work should focus on understanding the role of anti-inflammatory re-balancing factors of the IL-1 family, both for untangling the mechanisms in play behind these diseases, and in devising more specific approaches to such difficult patients.

## Figures and Tables

**Table 1:** Simplified scheme listing the most studied autoinflammatory disorders involving interleukin-1, nuclear factor-κB/tumor necrosis factor, or interferon as their dominant active cytokine.

**Inflammasomopathies and disorders of interleukin (IL)-1 production**				
**Disease Acronym**	**Gene (Locus)**	**Basic Disease Mechanism**	**Clinical Signs during Flares**	**Main Treatment Studied**
CAPS	*NLRP3* (1q44)	NLRP3-inflammasome activation	Fevers, fatigue, cold- induced urticaria-like rash, arthralgia, knee deformity, chronic meningitis, sensorineural deafness	Anakinra, rilonacept, canakinumab
FMF	*MEFV* (16p13.3)	Pyrin inflammasome activation	Fevers, polyserositis, arthritides, erysipelas- like rash, orchitis, association with vasculitides	Colchicine, canakinumab, anakinra
TRAPS	*TNFRSF1A* (12p13.31)	Multiple mechanisms; accumulation of misfolded TNF receptors (or defective shedding of TNF receptors) leading to inflammasome activation	Fevers, migratory centrifugal cellulitis-like rash, monocytic fasciitis. arthritides, abdominal pain, polyserositis, conjunctivitis	Corticosteroids, canakinumab, etanercept
MKD	*MVK* (12q24.11)	Altered biosynthesis of nonsterol isoprenoids with increased RhoA activity and pyrin inflammasome activation	Fevers, heterogeneous rash, abdominal pain, arthralgia, lymph node enalergement	On-demand anakinra, canakinumab
PAPAs	*PSTPIP1* (15q24.3)	Abnormal interaction with the pyrin inflammasome	Pyogenic arthritis, pyoderma gangrenosum, cystic acne	Corticosteroids, TNF-inhibitors, anakinra, canakinumab
MS	*LPIN2* (18p11.31)	Abnormal NLRP3 inflammasome activation	Chronic multifocal osteomyelitis, dyserythropoietic anemia, neutrophilic dermatosis	Anakinra, corticosteroids, bisphosphonates, TNF-inhibitors
AIEC	*NLRC4* (2p22.3)	NLRC4-inflammasome activation	Early-onset enterocolitis, arthralgia, pancytopenia, rash	Anakinra
DIRA	*IL1RN* (2q14.1)	Unbalanced IL-1 signaling	Neonatal-onset severe rash, multifocal osteomyelitis	Anakinra
**Disorders of nuclear factor (NF)-κB and/or aberrant activity of tumor necrosis factor (TNF)**				
**Disease acronym**	**Gene (locus)**	**Basic disease mechanism**	**Clinical signs**	**Main treatment studied**
BS/EOS	*NOD2/CARD15* (16q12.1)	NF-κB activation	Symmetric granulomatous arthritides, joint contractures, granulomatous panuveitis, granulomatous rash	Corticosteroids, methotrexate, TNF- inhibitors (infliximab, adalimumab), canakinumab
EOIBD	*IL10RA* (11q23.3), *IL10RB* (21q22.11), *IL37* (2q14.1)	Multiple mechanisms in play; aberrant IL-10 activity	Infantile ulcerative colitis	Corticosteroids, TNF-inhibitors (adalimumab), IL-18 inhibitors
DADA2	*CECR1* (22q11.1)	Dysregulation of TNF	Early-onset vasculitis, stroke-like disease, livedo reticularis	TNF-inhibitors (etanercept), tocilizumab
ORAS	*OTULIN* (5p15.2)	TNF-driven inflammation	Neonatal-onset fever, neutrophilic rash, panniculitis	TNF-inhibitors
A20h	*TNFAIP3* (6q23.3)	Dysregulation of NF-κB signaling	Oral and genital ulcers (familial Behçet-like disease), rash, gastrointestinal signs	TNF-inhibitors
RELAh	*RELA* (11q13.1)	Dysregulation of NF-κB signaling with IFN overproduction	Mucocutaneous ulcers, cytopenia, inflammatory bowel disease	TNF-inhibitors
HOIL-1d	*RBCK1* (20p13)	Abnormal NF-κB activation	Bacterial infections, lymphangectasia, colitis, muscular amylopectinosis	LUBAC inhibitors
TBK1d	*TBK1* (12q14.2)	TNF-driven inflammation	Arthritides, vasculitides	TNF-inhibitors
CRIAs	*RIPK1* (6p25.2)	IL-6 and TNF-driven inflammation	Fevers, gastrointestinal signs, arthralgia, oral ulcers, lymph node enlargement	Tocilizumab
**Interferon (IFN)-opathies**				
**Disease acronym**	**Gene (locus)**	**Basic disease mechanism**	**Clinical signs**	**Main treatment studied**
AGS	*TREX1* (3p21.31), *RNASEH2B* (13q14.3), *RNASEH2C* (11q13.1), *RNASEH2A* (19p13.13), *SAMHD1* (20q11.23), *ADAR* (1q21.3), *IFIH1* (2q24.2)	Abnormal nucleic acid processing and degradation	Subacute encephalopathy, cerebral calcifications, brain atrophy	None effective
SAVI	*TMEM137* (5q31.2)	Activation of the IFN I pathway	Interstitial lung disease, severe skin signs, distal ulcerative lesions with gangrene	Janus kinase inhibitors
COPas	*COPA* (1q23.2)	Activation of the IFN I pathway	Early-onset lung disease, arthritides, glomerulonephritis	Janus kinase inhibitors
PRAAS	*PSMA3* (14q23.1), *PSMB4* (1q21.3), *PSMB8* (6p21.32), *PSMB10* (16q22.1), *POMP* (13q12.3), *PSMG2* (18p11.21)	Subverted proteasome processing	Early-onset skin signs, neutrophilic dermatosis, joint contractures, lipodystrophy, panniculitis, myositis	Corticosteroids, anakinra, tocilizumab, TNF-inhibitors, Janus kinase inhibitors
NOCARH	*CDC42* (1p36.12)	Activation of the IFN I pathway, upregulation of pyrin-inflammasome, actin polymerization abnormality	Neonatal-onset lymphohistiocytosis, urticaria-like rash	Anakinra, Janus kinase inhibitors

Acronyms: AGS = Aicardi–Goutières syndrome; AIEC = autoinflammation with infantile enterocolitis; BS = Blau syndrome; CAPS = cryopyrin-associated periodic syndrome; COPas = COPA (coatomer protein a) syndrome; CRIAs = cleavage-resistant RIPK1-induced autoinflammation syndrome; DADA2 = deficiency of adenosine deaminase 2; DIRA = deficiency of interleukin-1 receptor antagonist; EOIBD = early-onset inflammatory bowel disease; EOS = early-onset sarcoidosis; FMF = familial Mediterranean fever; A20h = haploinsufficiency of A20; HOIL-1d = HOIL-1 deficiency; MKD = mevalonate kinase deficiency; MS = Majeed syndrome; NOCARH = neonatal-onset cytopenia, autoinflammation, rash and hemophagocytic lymphohistiocytosis; ORAS = OTULIN-related autoinflammatory syndrome; PAPAs = pyogenic arthritis pyoderma gangrenosum and acne syndrome; PRAAS = proteasome-associated autoinflammatory syndrome; RELAh = haploinsufficiency of RELA; SAVI = STING-associated vasculopathy with onset in infancy; TBK1d = TBK1 deficiency; TRAPS = tumor necrosis factor receptor-associated periodic syndrome.
